# Antibacterial and Antibiofilm Activity of Methanolic Plant Extracts against Nosocomial Microorganisms

**DOI:** 10.1155/2016/1572697

**Published:** 2016-06-27

**Authors:** Eduardo Sánchez, Catalina Rivas Morales, Sandra Castillo, Catalina Leos-Rivas, Ledy García-Becerra, David Mizael Ortiz Martínez

**Affiliations:** ^1^Departamento de Química, Facultad de Ciencias Biológicas, Universidad Autónoma de Nuevo León, Ciudad Universitaria, 66451 San Nicolás de los Garza, NL, Mexico; ^2^Departamento de Alimentos, Facultad de Ciencias Biológicas, Universidad Autónoma de Nuevo León, Ciudad Universitaria, 66451 San Nicolás de los Garza, NL, Mexico

## Abstract

Biofilm is a complex microbial community highly resistant to antimicrobials. The formation of biofilms in biotic and abiotic surfaces is associated with high rates of morbidity and mortality in hospitalized patients. New alternatives for controlling infections have been proposed focusing on the therapeutic properties of medicinal plants and their antimicrobial effects. In the present study the antimicrobial and antibiofilm activities of 8 methanolic plant extracts were evaluated against clinical isolated microorganisms. Preliminary screening by diffusion well assay showed the antimicrobial activity of* Prosopis laevigata*,* Opuntia ficus-indica*, and* Gutierrezia microcephala*. The minimum inhibitory concentration (MIC) and minimum bactericidal concentration (MBC) were determined ranging from 0.7 to >15 mg/mL. The specific biofilm formation index (SBF) was evaluated before and after the addition of plant extracts (MBC × 0.75).* Opuntia ficus-indica* caused the major reduction on SBF in dose-dependent manner. Cytotoxic activity of plant extracts was determined using brine shrimp lethality test (*Artemia salina *L.). Lethal Dose concentration (LD_50_ values) of the plant extracts was calculated. LD_50_ values for* P. laevigata* and* G. microcephala* were 141.6 and 323.3 *µ*g/mL, respectively, while* O. ficus-indica* showed a slight lethality with 939.2 *µ*g/mL. Phytochemical analyses reveal the presence of flavonoids, tannins, and coumarines.

## 1. Introduction

Microbial biofilms are communities of bacteria, embedded in a self-producing matrix, forming on living and nonliving solid surfaces [1]. Biofilm-associated cells have the ability to adhere irreversibly on a wide variety of surfaces, including living tissues and indwelling medical devices as catheters, valves, prosthesis, and so forth [2].

They are considered an important virulence factor that causes persistent chronic and recurrent infections; they are highly resistant to antibiotics and host immune defenses [3]. Bacteria protected within biofilm exopolysaccharides are up to 1,000 times more resistant to antibiotics than planktonic cells (free-floating) [4], which generates serious consequences for therapy and severely complicates treatment options [5]. An estimated 75% of bacterial infections involve biofilms that are protected by an extracellular matrix [6].

Biofilm resistance is due to several reasons, like restricted diffusion of antibiotics into biofilm matrix, expression of multidrug efflux pumps, type IV secretion systems, decreased permeability, and the action of antibiotic-modifying enzymes [7]. The increased biofilm resistance to conventional treatments enhances the need to develop new control strategies [8].

Biofilm inhibition is considered as major drug target for the treatment of various bacterial and fungal infections, and pharmacological development of this drugs is now extensively studied [9]. In recent years, several green nonlethal strategies for biofilm control have been developed, because the mode of action of these novel antibiofilm agents is much less susceptible to the emergence of resistance [10]. However although they are promising strategies, they have disadvantages because none have been totally effective [5].

One promising alternative is the search for naturally occurring compounds of plant origin capable of blocking biofilm formation [11]. Historically, plant extracts and their biologically active compounds have been a valuable source of natural products, which have played a central role in the prevention and treatment of diseases, helping to maintain human health [12]. Furthermore, they are widely accepted due to the perception that they are safe and have a long history of use in folk medicine to cure diseases and illnesses since ancient times [13].

Considering the above and based on previous results obtained in our laboratory, in the present study we propose to evaluate the antibiofilm effect of 8 extracts plants against 5 clinical isolated pathogens.

## 2. Material and Methods

### 2.1. Plant Material

Fresh and healthy plants growing wild around the Casablanca community, located in Santa Catarina, Nuevo León, Mexico (25°39′11.33′′N 100°42′41.09′′W), were collected between March and April 2014. Voucher samples were deposited at the herbarium of the Botanical Department of Biological Sciences School, Universidad Autónoma de Nuevo León, for identification purposes. Collected plants (Table 1) were washed thoroughly in tap water, followed by successive washing in distilled water. Washed plants were cut into small pieces and air-dried at room temperature (25 ± 2°C) under shade. Finally, dried material was grounded to coarse powder in a manual grain mill and stored in plastic containers for further analysis.

### 2.2. Bacterial Strains and Culture Conditions

The microorganisms used in this study were 5 nosocomial pathogens, 4 Gram-negative (*Klebsiella pneumoniae*,* Enterococcus faecalis*,* Escherichia coli*, and* Stenotrophomonas maltophilia*) and 1 Gram-positive (*Staphylococcus aureus*). All strain were kindly provided by Dra. Elvira Garza Gomzález, School of Medicine, UANL. Strains were maintained in Mueller-Hinton (MH) agar (Difco) at 4°C. Active cultures were obtained by inoculation of a loopful of each strain into separated 5 mL MH broth (Difco) and incubated for 18 h at 37°C.

### 2.3. Preparation of Plant Extracts

Extracts were prepared following the methodology proposed by Sánchez et al. [14], with minor modifications. Briefly, one hundred grams (100 g) of dried plant material was soaked with 500 mL of methanol for 24 h at room temperature (25 ± 2°C), under occasional shaking. Extraction was repeated three times, and the extracts obtained were combined and filtered through Whatman filter paper number 1. After that, they were concentrated to dryness under reduced pressure using a rotary evaporator at 45°C. Stock solutions (200 mg/mL) were prepared in methanol and stored at 4°C in the dark for further experiments.

### 2.4. Qualitative Phytochemical Screening

The extracts were subjected to standard phytochemical tests in order to evaluate their chemical composition for different active constituents; for this extracts (3–5 mg/mL) they were separately suspended in 1 mL of absolute ethanol or distilled water (carbohydrate determination) using clean test tubes.

### 2.5. Bayer's Test for Unsaturation

In this case aqueous 1% KMnO_4_ was added dropwise to the extract solution. A positive test was evidenced by the disappearance of the purple color of KMnO_4_ and the appearance of a brown solid precipitate (MnO_2_) [15].

### 2.6. Detection of Triterpenes/Steroids (Liebermann-Burchard Reagent)

One mL of acetic anhydride and 5 drops of concentrated sulfuric acid (H_2_SO_4_) were added to the extract. A color change from violet to blue confirms the presence of steroids [16] and formation of blue-green ring indicated the presence of terpenoids [17].

### 2.7. Coumarins

Three mL of 2 N NaOH was added to 2 mL of aqueous extract. Formation of yellow color indicated the presence of coumarins. Confirmation test was performed by adding 1 mL of 5 N HCl; in this case a colorless solution formed at the upper layer is considered positive [18].

### 2.8. Alkaloids

Ethanolic extracts (20 *μ*L) were applied on TLC plates (Silica Gel 60G, 5 × 10 cm) and eluted using toluene-ethyl acetate-diethylamine (70 : 20 : 10) as solvent system. Alkaloids were detected after spraying Dragendorff's reagent as orange-brown spots on TLC plates [19].

### 2.9. Screening for Sesquiterpene Lactones

The Baljet reaction (1% Picric acid in 10% sodium hydroxide) was used to detect sesquiterpene lactones in the extracts. Reagents were mixed at a 1 : 1 ratio and added to 1 mL of extracts (2-3 mg). The transformation of the sodium picrate solution's yellow color to orange-red color confirmed the positive reaction [20].

### 2.10. Test for Quinones

Extracts suspended in ethanol (1 mL) were treated with 1 mL of concentrated sulfuric acid. Formation of red color shows the presence of quinones [21].

### 2.11. Carboxyl Group

The presence of carboxyl groups was evidenced by adding 10 drops of 10% sodium bicarbonate solution; visible bubbles of carbon dioxide were considered a positive reaction [21].

### 2.12. Test for Tannins

Extracts were treated with 1 mL of 5% ferric chloride which was added. The presence of tannins was indicated by the formation of bluish black or greenish black precipitate [22].

### 2.13. Shinoda Test

Few fragments of magnesium metal ribbon (3-4 pieces) were added to 1 mL of ethanolic extract, followed by dropwise addition of concentrated hydrochloric acid. Formation of pink or red color indicated the presence of flavonoids [23].

### 2.14. Saponin

Two mL of distilled water was added to extracts suspended in ethanol and was shaken vigorously. The formation of copious foam layer indicates the presence of saponins [23].

### 2.15. Carbohydrates

For carbohydrates test, extracts (10 mg) were suspended in 1 mL of distilled water; afterward 2 mL of 0.2% anthrone reagent and 5 drops of concentrated sulfuric acid were added. Dark green color showed the presence of carbohydrates [21].

### 2.16. Evaluation of Antimicrobial Activity

Antimicrobial activity of plant extracts was performed using the agar-well diffusion bioassay. Briefly, 100 *μ*L of fresh culture (approximately 10^6^ CFU/mL) was uniformly spread onto Mueller-Hinton agar (MHA) plates by sterile Driglasky loop. Then, inoculated plates were allowed to dry at room temperature for 20 min. After that, wells of 6 mm in diameter were made in the agar using a sterilized cup-borer and 100 *μ*L of each extract was poured in the wells. Methanol was used as control. Plates were incubated at 37°C for 18 h. Antibacterial activity was evidenced by the presence of clear inhibition zone around each well. The diameter of this zone was measured and recorded [14].

### 2.17. Assessment of Minimum Inhibitory Concentration (MIC) and Minimum Bactericidal Concentration (MBC)

The MIC and MBC were determined on plant extracts that showed antimicrobial activity, by a broth microdilution method proposed by Novy et al. [24], with minor modifications. Briefly, 100 *μ*L of Mueller-Hinton Broth (Difco) plus different concentrations of plant extracts was prepared and transferred to each microplate well to obtain dilutions of the active extract, ranging from 1.0 to 25 mg/mL. Then, 10 *μ*L of a fresh culture (final concentration of 1 × 10^6^ CFU/mL) of test organisms was added. Microplates were incubated at 37°C for 24 h [25]. MIC was defined as the lowest concentration of the extract that restricted the visible growth of microorganism tested.

To determine MBC, 100 *μ*L from each well that showed no visible growth was reinoculated on MH agar plates; then the plates were incubated at 37°C for 24 h. MBC was defined as the lowest extract concentration showing no bacterial growth. Methanol was used as blank and tetracycline (Sigma Aldrich, Mexico City, Mexico) as positive control. Once the MBC was recorded, the sublethal activity on bacterial growth was determined; for this, concentrations of 75, 50, and 25% of MBC were tested in a 96-well microplate and the counts of microbial cells were done by plate count technique, as previously mentioned.

### 2.18. Biofilm Formation Inhibition

The effect of extracts on biofilm formation was evaluated in 96-well polystyrene flat-bottom plates [26]. Briefly, 300 *μ*L of inoculated fresh trypticase soy yeast broth (TSY) (final concentration 10^6^ CFU/mL) was aliquoted into each well of microplate and cultured in presence of sublethal concentrations (75, 50, and 25% of MBC) previously determined. Wells containing medium and those without extracts and only with methanol were used as controls. Plates were incubated at 37°C for 48 h. After incubation, supernatant was removed and each well was washed thoroughly with sterile distilled water to remove free-floating cells; thereafter plates were air-dried for 30 min and the biofilm formed was stained during 15 min at room temperature with 0.1% aqueous solution of crystal violet. Following incubation, the excess of stain was removed washing the plate three times with sterile distilled water. Finally, the dye bound to the cells was solubilized by adding 250 *μ*L of 95% ethanol to each well and after 15 min of incubation, absorbance was measured using microplate reader at a wavelength of 570 nm. Biofilm determination was made using the formula SBF = (AB − CW)/G, where SBF is the specific biofilm formation, AB is the OD570 nm of the attached and stained bacteria, CW is the OD570 nm of the stained control wells containing only bacteria-free medium, and G is the OD630 nm of cell growth in broth [27].

### 2.19. Toxicity Bioassay

Brine shrimp (*Artemia salina*) lethality bioassay was carried out in accordance with methodology proposed by Meyer et al. [28] to determine the toxicity of extract plants. For this, brine shrimps cysts were hatched in a shallow rectangular container, which was divided into two unequal compartments, filled with sterile artificial seawater (prepared by dissolving sea salt 38 g/L and adjusted to pH 8.5 using 1 N NaOH) under constant aeration and proper light. Cyst (ca. 50 mg) was sprinkled into the larger compartment, which was darkened, while the smaller was illuminated. Yeast solution 0.06% was added to the hatching chamber to feed the larvae. After 48 h the phototropic free nauplii were collected from the lighted side.

Lethality bioassay was performed using 10 collected nauplii, which were transferred into vials contained tested crude plant extract, at 10, 100, and 1000 *μ*g/mL, and artificial seawater. Appropriate quantities of methanol were used as negative control.

After 24 h of incubation, live nauplii were counted and the LC_50_ values were estimated using a Probit regression analysis. Extracts giving LC_50_ values above 1000 *μ*g/mL were considered nontoxic.

### 2.20. Statistical Analysis

All experimental results were expressed as mean ± standard deviation (SD) for analysis performed in duplicate at least three times. Statistical analysis of the data was performed by Analysis of Variance (ANOVA) and mean comparison using Student's *t*-test, using SPSS software version 17.0. The LC_50_ for bioassay with* A. salina* was determined according to the Probit statistical method. *P* < 0.05 was considered statistically significant.

## 3. Results and Discussion

A total of 8 methanolic plant extracts were tested against 5 clinical bacterial isolates. Methanol was selected as extraction solvent, because it is one of the best solvents used for the extraction of antimicrobial substances [29, 30]. Moreover, methanol polarity ensured the extraction of polar and moderately polar active compounds from plants against microorganisms like terpenoids, tannins, flavones, and polyphenols [31].

Results of preliminary antimicrobial tests, performed by the well diffusion method, were quite variable between each plant extract ranging from 0 to 2.8 cm (Table 2).* P. laevigata* extract was active against all the clinical isolates, while* N. bivalve* bulb did not show activity against any microorganism. The highest diameter of inhibition was obtained with* P. laevigata* extract (2.8 ± 0.5 cm), against* S. aureus* strain, followed by* G. microcephala* (2.3 ± 0.2 cm) and* O. ficus-indica* (1.6  ±  0.3 cm) also against* S. aureus*. Meanwhile* E. coli* was less susceptible to these extracts showing diameters of 1.7 ± 0.3, 1.4 ± 0.1, and 1.6 ± 0.1 cm, respectively.* K. pneumoniae* and* E. faecalis* were more resistant to the extracts, only inhibited by* P. laevigata* and* O. ficus-indica* with inhibition zones ranging from 0.7 ± 0.08 to 1.3 ± 0.2; on the other hand,* S. maltophilia* was the only microorganism that was not inhibited by the extracts.

However, the well diffusion assay is considered a qualitative technique and is mainly used for selecting extracts with antimicrobial activity, mostly when diameters zones of inhibition are ≥10 mm [32]. It is important to recognize that the size of inhibition zones of different extracts could be due to the compounds polarity obtained, since a more diffusible but less active extract could give a bigger diameter of inhibition than a nondiffusible but more active extract [33].

Minimum inhibitory concentration (MIC) results are comparable to those obtained in the agar-well diffusion technique, because the lowest MIC were obtained using the extracts showing the best antimicrobial activity (data not shown). Meanwhile results of minimum bactericidal concentrations (MBC) are listed in Table 3, where* P. laevigata* extract had the lowest MBC with a value of 2 mg/mL for* E. coli*, 2.8 mg/mL for* E. faecalis*, 3.8 mg/mL for* K. pneumoniae*, and 0.7 mg/mL for* S. aureus*. Extracts and* O. ficus-indica* got the highest CMB ranging from 1.0 to ≥15 mg/mL. CMBs of* G. microcephala* were 2.8 and 8.3 mg/mL against* S. aureus* and* E. coli*, respectively. MBC results show that* S. aureus* was the more sensitive microorganism, being inhibited for 8 methanolic extracts, while* S. maltophilia* was not inhibited by any extract. Broadly, our results agree with previous reports, which mention greater activity of extracts towards Gram-positive microorganisms compared to Gram-negative microorganisms [34]. These differences can probably be attributed to the structural and compositional differences in the cell wall and membranes [25]. The Gram-negative bacteria have an outer membrane that serves as barrier for many molecules; also, the presence of efflux pump system has been demonstrated, which can mediate the resistance to natural compounds [35].* Escherichia coli* was the most susceptible of the Gram-negative bacteria; this finding also agrees with previous reports [36].

According to the previously mentioned results, it was decided to select 3 plant extracts (*P. laevigata*,* O. ficus-indica*, and* G. microcephala)* which were active against* E. coli* (Gram-negative) and* S. aureus* (Gram-positive); moreover these extracts showed the lowest MBC.

Phytochemical screening results of selected plant extracts are summarized in Table 4 and show the presence of different functional groups. Coumarins, alkaloids, tannins, and flavonoids were found in* P. laevigata* extract. Similar compounds have been reported in different species of this plant like* P. juliflora*, where the presence of tannins, phenolics, flavonoids, steroids, terpenes, and alkaloids has been reported [37]. Likewise, reports of* Prosopis* spp. mentioned that this plant contains harmine, prosopine which is an alkaloid reported in several papers, tyramine, prosopinine, and juliflorine, which are alkaloids that intercalate into DNA and could explain the antimicrobial activity of this extract [31, 38].

In case of* O. ficus-indica*, results indicate the presence of triterpenes, coumarins, quinones, tannins, carbohydrates, and flavonoids; flavonoids cause bacterial death by inhibiting DNA or RNA synthesis and tannins including possible inhibition of extracellular microbial enzymes [39, 40].

Meanwhile, triterpenes, coumarins, quinones, tannins, flavonoids, and sesquiterpene lactones were found in* G. microcephala*. According to Gören et al. [41] sesquiterpene lactones are the main secondary metabolite responsible for the antimicrobial activity in Asteraceae family. While McDaniel and Ross [42] report the presence of alkaloids and saponins conferring some toxicity at this plant.

**Figure 1:**
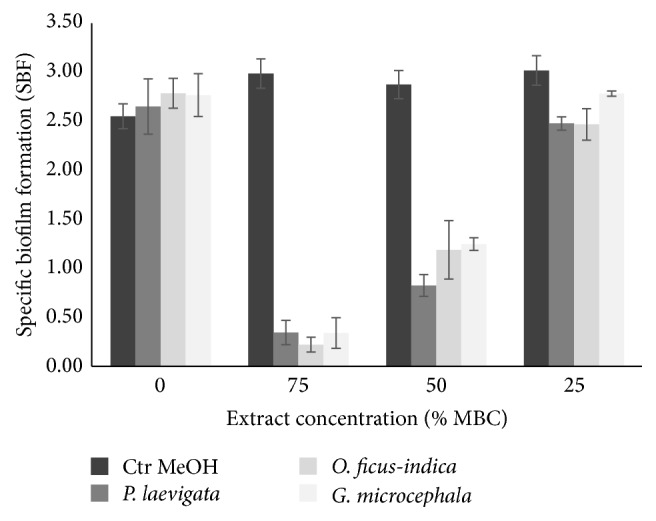
Inhibition of biofilm formation by different concentrations of plant extracts against* E. coli*.

Biofilm formation inhibition results by addition of subinhibitory concentrations (75, 50, and 25% of MBC) of plant extracts against* E. coli* and* S. aureus* indicated that the obtained effect was dose-dependent. The best biofilm reduction is observed in higher concentrations of the extracts (75% of WBC). Similar results were reported by Issac Abraham et al. [43], who reported that methanolic caper extract significantly inhibited biofilm formation and EPS production in* E. coli*,* Serratia marcescens*,* Pseudomonas aeruginosa*, and* Proteus mirabilis*. As well Ravichandiran et al. [44] reported that ethanolic extract of the bark of* Melia dubia* caused a strong suppression of hemolysis, swarming motility, and biofilm formation in* E. coli*. Results of the effect of concentrations corresponding to 75 and 50% of MBC caused significant (*P* < 0.05) reduction of the specific biofilm formation (SBF) of* E. coli* (Figure 1) from approximately 3 (strong biofilm) to levels of 0.2 (weak biofilm, 75% MBC) and 1.2 (moderated biofilm, 50% MBC). The SBF classification categories were mentioned by Mittal et al. [45] who mention that strong biofilm producers (SBF index > 2.00), intermediate biofilm producers (SBF index between 1 and 2), and weak biofilm producers (SBF index < 1.00). Similar results were obtained with* S. aureus* (data not shown). Inhibition of biofilm formation can be explained by the presence of flavonoids, previously reported such as quercetin, kaempferol, naringenin, and apigenin, which are capable of reducing biofilm synthesis because they can suppress the activity of the autoinducer-2 responsible for cell-to-cell communication [46].

*A. salina* bioassay is used to evaluate the toxicity of plant extracts and has the advantage of being inexpensive, reliable, and reproducible [47]. In a previous study, Ahmed et al. [48] determined the toxicity of methanol extract of* Prosopis spicigera* reporting 60% survived nauplii at 100 *μ*g/mL which is consistent with the results obtained in this work, because LD_50_ obtained of* P. laevigata* was 141.6 *μ*g/mL indicating that the extract is moderately toxic; this may be due to the presence of certain bioactive compounds which may be related to the antibacterial activity. For* G. microcephala* was moderately toxic with LD_50_ of 323.3 *μ*g/mL, some studies mentioned that this toxicity may be due to the presence of saponins, essential oils, mono- and sesquiterpenes, tannins, and alkaloids [42, 49]. Results of* O. ficus-indica* indicate slight toxicity (939.2 *μ*g/mL); this is consistent as reported by Déciga-Campos et al. [50]. Low toxicity could be explained with the common use of this plant in traditional medicine. Furthermore, in vivo and in vitro experiments of cladodes and fruits show a beneficial effect on health due to the presence of flavonoids, which have health-related properties, which are based in their antioxidant activity [51, 52].

## 4. Conclusions

Some of the plant extracts evaluated in present research had potential antimicrobial and antibiofilm activities against isolated nosocomial bacteria, which can be an alternative to control the formation of microbial biofilms or can be used as model to the search for new drugs.

## Figures and Tables

**Table 1:** Overview of the collected plants used in this investigation.

Scientific name	Common name	Family	Part used	Voucher number
*Sophora secundiflora* (Ortega) Lag. Ex DC.	Mountain laurel	Fabaceae	Aerial parts	027770
*Sphaeralcea ambigua* A. Gray	Desert globemallow	Malvaceae	Bark	027771
*Prosopis laevigata* (Humb. et Bonpl. ex Willd) M.C. Johnston	Smooth mesquite	Fabaceae	Bark and leaves	027772
*Opuntia ficus-indica* Mill.	Nopal cactus	Cactaceae	Cladode	027773
*Marrubium vulgare* L.	White horehound	Lamiaceae	Aerial parts	027774
*Scutellaria drummondii* Benth	Drummond's skullcap	Lamiaceae	Aerial parts	027775
*Nothoscordum bivalve* Britton.	Crowpoison	Alliaceae	Bulb	027776
*Gutierrezia microcephala* (DC.) Gray	Sticky snakeweed	Asteraceae	Aerial parts	027777

**Table 2:** Diameter of inhibition zone of methanolic extracts against clinical isolated bacteria.

Plant	Inhibition zone (cm)				
	*K. pneumoniae*	*E. faecalis*	*E. coli*	*S. maltophilia*	*S. aureus*
*S. secundiflora*	NI	NI	NI	NI	2.1 ± 0.3
*S. ambigua*	NI	NI	NI	NI	1.2 ± 0.1
*P. laevigata*	1.4 ± 0.3	1.7 ± 0.3	1.5 ± 0.3	NI	2.6 ± 0.3
*O. ficus-indica*	1.7 ± 0.1	1.5 ± 0.1	1.6 ± 0.3	NI	1.6 ± 0.3
*M. vulgare*	NI	0.7 ± 0.01	NI	NI	1.8 ± 0.2
*S. drummondii*	NI	0.6 ± 0.01	NI	NI	1.7 ± 0.2
*N. bivalve *	NI	NI	NI	NI	NI
*G. microcephala*	NI	NI	1.6 ± 0.1	NI	2.3 ± 0.2

Values are means ± standard deviations. NI: no inhibition.

**Table 3:** Minimum bactericidal concentration (MBC) of methanolic extracts against clinical isolated bacteria.

Plant	MBC (mg/mL)				
	*K. pneumoniae*	*E. faecalis*	*E. coli*	*S. maltophilia*	*S. aureus*
*S. secundiflora*	NE	NE	NE	NE	9.1 ± 0.4
*S. ambigua*	NE	NE	NE	NE	>15
*P. laevigata*	3.8 ± 0.1	2.7 ± 0.1	1.5 ± 0.2	NE	0.7 ± 0.01
*O. ficus-indica*	>15	>15	4.0 ± 0.3	NE	1.0 ± 0.2
*M. vulgare*	NE	0.7 ± 0.01	NE	NE	3.9 ± 0.3
*S. drummondii*	NE	0.6 ± 0.01	NE	NE	7.3 ± 0.2
*N. bivalve *	NE	NE	NE	NE	NE
*G. microcephala*	NE	NE	8.3 ± 0.2	NE	2.8 ± 0.3

Values are means ± standard deviations. NE: not evaluated.

**Table 4:** Phytochemical screening results of selected methanolic extracts.

Compounds	*P. laevigata*	*O. ficus-indica*	*G. microcephala*
Unsaturation	—	++	++
Triterpenes/steroids	++/Steroids	++/Triterpenes	+++/Triterpenes
Coumarins	+++	+	+++
Alkaloids	+++	—	—
Sesquiterpene lactones	—	—	++
Quinones	—	+	+
Carboxyl group	—	—	—
Tannins	+++	++	+++
Saponins	—	—	—
Carbohydrates	++	+++	—
Flavonoids	++	+	++

+: low intensity reaction, ++: medium intensity reaction, and +++: strong intensity reaction.
